# Gas Gangrene as Seen at the Casualty Stations

**Published:** 1917-12

**Authors:** 


**Gas Gangrene as Seen at the Casualty Stations.** Cont. of abs. from p. 109 of the article by CUTHBERT S. WALLACE.

*Conclusions as Regards Treatment.*

*a*) W. insists upon the avoidance of all pressure or other hindrance to circulation, and recommends especially that all hemorrhages and hematomae be hunted out and corrected. In cases involving the injury or thrombosis of great vessels, he urges that an attempt be made to suture rather than resort to ligature. In this respect Tuffier's tube may be serviceable,

*b*) In considering amputation it is well to remember that only the wounded muscle is likely to be infected with gaseous gangrene, and that the excision or the ablation of this muscle usually suffices to arrest infection. This is not so easily accomplished, however, in the thigh as in the leg, in which case it is fairly easy to save the limb by the ablation of the anterior tibial group. The same holds true for the muscles of the fore-arm. The brick red color and the non-contractibility will show at once which muscles are past saving.

*c*) When gas gangrene occurs in a segment of a limb distal to the segment wounded it nearly always means that the main artery is blocked and amputation of the gangrenous segment is the only course.

*d*) W. warns against taking the extent of crepitation of the skin as an indication for amputation, for it may not necessarily indicate a state of infection requiring such drastic treatment. The surgeon before deciding should determine accurately the exact condition of the muscles and the number involved. Otherwise many limbs may be sacrificed when the removal of only a single muscle might serve to check the infection.

